# Higher oxidative balance score is linearly associated with reduced prevalence of chronic kidney disease in individuals with metabolic syndrome: evidence from NHANES 1999–2018

**DOI:** 10.3389/fnut.2024.1442274

**Published:** 2024-09-30

**Authors:** Linying Zhu, Xiaoyi Ruan, Jianqi Wang, Yongxing Yan, Chunyuan Tang, Yuanwen Xu

**Affiliations:** ^1^School of Obstetrics and Pediatrics, Guangdong Medical University, Zhanjiang, Guangdong, China; ^2^The First Clinical College, Guangdong Medical University, Zhanjiang, Guangdong, China; ^3^Department of Nephrology, The First Affiliated Hospital of Sun Yat-sen University, Guangzhou, Guangdong, China

**Keywords:** oxidative balance score, oxidative stress, metabolic syndrome, chronic kidney disease, NHANES

## Abstract

**Background:**

Oxidative stress is a key contributor to the development of chronic kidney disease (CKD) in individuals with metabolic syndrome (MetS). The oxidative balance score (OBS) is an emerging composite assessment tool for dietary and lifestyle oxidative balance. We aimed to explore the association of OBS with CKD prevalence in MetS in this national cross-sectional analysis.

**Methods:**

This was a national cross-sectional analysis. Eligible MetS participants ≥20 years of age from NHANES 1999–2018 were included. OBS was assessed according to previous well-validated methods and consisted of 16 dietary components and 4 lifestyle components. MetS was diagnosed by NCEP-ATP III criteria, while CKD was diagnosed by KDIGO 2021 Clinical Practice Guideline. Multivariate logistic regression models were used to explore the association of OBS with CKD in MetS in this national cross-sectional analysis.

**Results:**

A total of 8,095 MetS participants were included, with a CKD prevalence of 24.8%. In fully adjusted models, each score increases in OBS, dietary OBS, and lifestyle OBS was associated with a 2, 1.7, and 7.3% reduction in the prevalence of CKD, respectively. Higher OBS, dietary OBS, and lifestyle OBS were all associated with significantly lower odds of CKD (*p* for trend all <0.05). Restricted cubic spline analysis showed that these associations all exhibited inverse dose–response. Interaction analyses indicated that cardiovascular disease (CVD) status significantly influenced the impact of OBS and dietary OBS, and these associations were only present in CVD-free subjects. Defining MetS using the IDF criteria did not significantly change the results.

**Conclusion:**

OBS was inversely associated with the prevalence of CKD in MetS, especially in CVD-free settings. These findings emphasize that adherence to an antioxidant diet and lifestyle contributes to the early prevention of CKD in the MetS population and necessitates attention to CVD interactions. Future prospective cohort studies are needed to confirm these results.

## 1 Introduction

Metabolic syndrome (MetS) refers to a cluster of cardiovascular risk factors characterized by abdominal obesity, insulin resistance (IR), hypertension, and hyperlipidemia (1). MetS is a major global public health concern and is one of the most common non-communicable chronic conditions worldwide, affecting about a quarter of the world’s population (2). In addition, the global burden of MetS is still increasing due to the prevalence of obesity and type 2 diabetes (T2D) caused by unhealthy lifestyles (3). MetS is not a separate disease *per se*, but its clinical significance lies in its close association with the development of a variety of other major cardiometabolic disorders, including cardiovascular disease (CVD), T2D, non-alcoholic fatty liver disease, multiple cancers, and chronic kidney disease (CKD) (4–8). CKD is considered as one of the major complications of MetS, which is thought to have substantial crosstalk and share many common pathogenic mechanisms with metabolic disorders and CVD, i.e., the Cardiovascular-Kidney-Metabolic (CKM) Syndrome recently proposed by the American Heart Association (9–11). Numerous epidemiologic studies have demonstrated a significant bidirectional relationships between MetS and CKD (12). The co-morbidity of CKD in MetS is associated with significantly increased risk of comorbidities and mortality, severely impairing individuals’ health-related quality of life and placing a heavy burden on society (13). Identifying modifiable risk factors for the development of CKD in MetS and undertaking interventions has important public health implications for reducing the MetS disease burden.

Although the mechanisms and pathways involved in CKD pathogenesis in MetS remain incompletely understood, accumulating evidence suggests that oxidative stress is a major contributor. The main hallmarks of MetS including IR and visceral obesity can induce systemic chronic low-grade inflammation and oxidative stress, leading to microvascular remodeling in the kidney thus leading to CKD onset and progression (14, 15). Exogenous antioxidants and pro-oxidants have been implicated as potentially modulating the individual’s level of oxidative stress and influencing the development of disease (16). However, most of the previous studies have only explored the association of specific dietary antioxidants or pro-oxidants with MetS or CKD, with shortcomings in reflecting the overall dietary and lifestyle oxidative balance of the individual (17, 18).

To comprehensively measure an individual’s antioxidant and pro-oxidant exposure, the oxidative balance score (OBS) has been proposed as an integrated assessment tool (19). OBS provides a global appraisal of individual dietary and lifestyle exposures to antioxidants and pro-oxidants. An antioxidant diet and lifestyle are suggested to modulate the body’s redox balance and reduce the risk of a range of diseases, including CKD (20, 21). However, the association between OBS and CKD in MetS remains poorly studied, especially given the heavy disease burden and interactions of CKD in MetS. In addition, previous studies have some limitations that need to be addressed. Previous studies may lack comprehensiveness due to the small number of OBS components included and require further updating. The respective effects of dietary OBS and lifestyle OBS have not been explored previously, thus preventing individualized prevention strategies from being derived. Finally, there is a lack of adequate exploration of the interactions between metabolic disorders, CVD, and CKD.

Therefore, to address the current knowledge gap, we aimed to explore the association of OBS with CKD in MetS through a nationally representative, population-based, ongoing large cross-sectional survey, the National Health and Nutrition Examination Survey (NHANES). These findings may underpin the prevention of CKD in individuals with MetS who are adherent to antioxidant diets and lifestyles, thereby reducing disease burden and improving prognosis.

## 2 Methods

### 2.1 Study design and population

NHANES is the primary program conducted by the National Center for Health and Statistics (NCHS), part of the Centers for Disease Control and Prevention, to assess the health and nutritional status of non-institutionalized citizens in the U.S. Beginning in 1999, NHANES has been conducted in a two-year survey cycle, in which approximately 5,000 nationally representative participants are drawn from across the U.S. each year. NHANES is a series of ongoing, nationally representative cross-sectional surveys with a complex multi-stage probability sampling cluster design.

**Figure 1:**
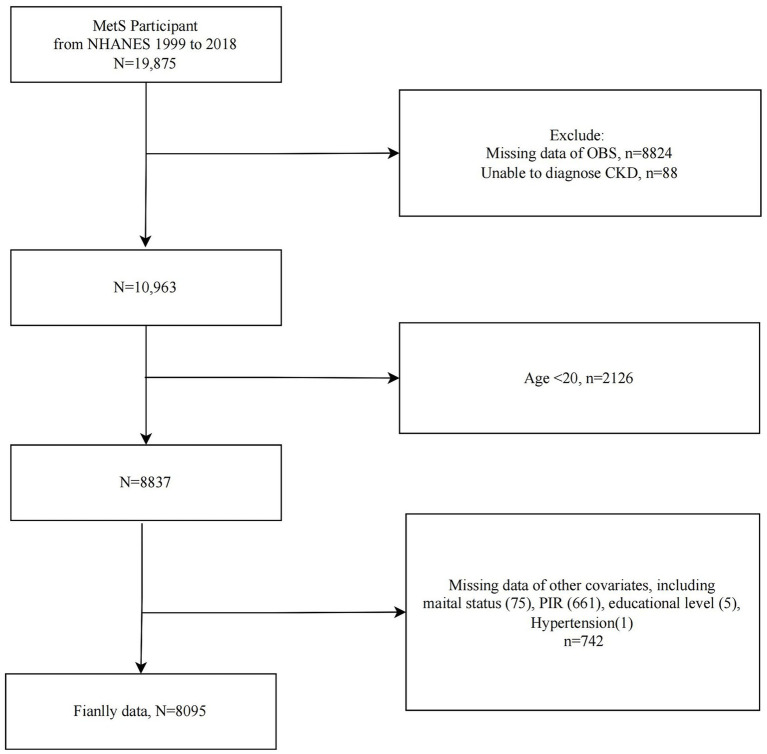
Flowchart of study population selection, NHANES 1999–2018.

The study population selection flowchart was presented in Figure 1. We included MetS participants from 10 cycles of NHANES 1999–2018 (*n* = 19,875) and sequentially excluded those with missing OBS information (*n* = 8,824), CKD diagnosis information (*n* = 88), participants <20 years of age (*n* = 2,126), and those with missing covariate data (*n* = 742). Ultimately, 8,095 eligible MetS participants were included for further analysis.

### 2.2 Evaluation of OBS

The components and assignment criteria for OBS have been extensively discussed in previous studies using NHANES similarly (22, 23), and we adopted the well-established methodology from previous studies for OBS assessment and presented in Supplementary Table S1. Briefly, the OBS consists of 16 dietary components and 4 lifestyle components. Dietary OBS consists of dietary sources of 14 antioxidants (dietary fiber, carotene, riboflavin, niacin, Vitamin B6, total folate, Vitamin B12, Vitamin C, Vitamin E, calcium, magnesium, zinc, copper, and selenium) and 2 pro-oxidants (total fat and Iron). Lifestyle OBS consisted of 1 antioxidant (physical activity) and 3 pro-oxidants [body mass index (BMI), alcohol consumption, and cotinine exposure]. The mean of two 24-h dietary recall surveys was used to represent their intake (the first was a 24-h dietary recall interview conducted at the mobile examination center, and a telephone follow-up 3–10 days later). Physical activity was assessed according to metabolic equivalents [MET, min/week]. Alcohol intake data were obtained from dietary recalls, where intakes greater than 30/15 g/d for men/women, respectively, were suggestive of significant alcohol consumption. BMI was calculated based on weight (kg) divided by the square of height (m). Serum cotinine exposure was used as a combined proxy for the presence of active and passive smoking. Overall, the OBS assigns scores to antioxidant (0, 1, and 2 points, respectively, on a low-to-high scale) and pro-oxidant (2, 1, and 0 points, respectively, on a low-to-high scale) levels based on gender-specific criteria. Most of the components were grouped according to tertiles (except for the previously mentioned alcohol consumption).

### 2.3 Assessment of MetS

The National Cholesterol Education Program-Adult Treatment Panel III criteria were used to assess MetS (24). MetS was defined as meeting ≥3 of the following five criteria: (1) Waist circumference (WC) ≥102 cm in men and ≥88 cm in women; (2) Serum triglycerides (TG) ≥150 mg/dL; (3) Serum high-density lipoprotein (HDL) cholesterol (HDL-C) <40 mg/dL in men or <50 mg/dL in women; (4) Fasting blood glucose (FBG) ≥100 mg/dL or use of hypoglycemic drugs; (5) Blood pressure (BP) ≥130/85 mmHg. Information on serum TG, HDL-C, and FBG was obtained from the biochemical test files at NHANES, while BP was obtained from three consecutive measurements taken by professionals at the mobile examination center. In the sensitivity analysis, we assessed MetS using another widely used diagnostic criterion, the International Diabetes Federation (IDF) (25), to verify the consistency of the results.

### 2.4 Diagnosis of CKD

The CKD was defined as having a urinary albumin/creatinine ratio (uACR) ≥ 30 mg/g and/or an estimated glomerular filtration rate (eGFR) < 60 mL/min/1.73 m^2^ (26). eGFR was calculated according to the widely accepted Chronic Kidney Disease Epidemiology Collaboration formula (27). Urine albumin was measured by fluorescence immunoassay and urine creatinine was measured by enzymatic method. Urine albumin and creatinine levels were standardized and calibrated in NHANES using gold standard methods.

### 2.5 Covariates

We included several potential covariates, including age, sex (male/female), race/ethnicity (Mexican American/non-Hispanic White/non-Hispanic Black/other Hispanic/other race), education level (<high school/high school/>high school), income-poverty ratio (PIR), marital status (single/non-single), daily dietary energy intake, diabetes, hypertension, and CVD. Daily energy intake (kcal/day) was derived from self-reporting in face-to-face dietary recalls at mobile examination centers. Diabetes was indicated by one of the following: self-reported history of diabetes, fasting blood glucose ≥7 mmol/L, Hemoglobin A1c ≥ 6.5%, oral glucose tolerance test or random blood glucose ≥11.1 mmol/L, or current use of antidiabetic medications. The presence of hypertension was indicated by a self-reported history of hypertension, a blood pressure test value of ≥140/90 mmHg or being on antihypertensive medication. CVD history was obtained through participants’ affirmative responses to specific questions on the Medical Conditions Questionnaire.

### 2.6 Statistical analysis

We weighted all analyses appropriately according to NHANES analytic guidelines to account for the complex study design of NHANES and to enable nationally representative estimates of the sample. Data processing and analysis were performed using EmpowerStats (X&Y Solutions, Inc., Boston, MA, United States) and R software (version 4.2.3). Bilateral *p-*values of less than 0.05 were defined as statistically significant. In baseline analyses, participants were grouped according to OBS quartiles or CKD status, with continuous variables expressed as mean ± standard error and categorical variables expressed as number (percentage). Continuous variables were compared using weighted ANOVA or *t*-tests, and categorical variables were analyzed using chi-square tests. Multiple multivariate logistic regression models were used to explore the association between OBS and CKD in MetS and to calculate odds ratios [OR] and 95% confidence intervals [CI]. OBS, dietary OBS, and lifestyle OBS were considered either continuous or categorical variables (quartiles). Model 1 did not adjust for any covariates, model 2 partially adjusted for age, sex, and race, and model 3 additionally adjusted for education, PIR, marital status, daily energy intake, diabetes mellitus, hypertension, and CVD on top of model 2. Restricted cubic spline (RCS) was used to explore dose–response associations or potential non-linear associations. The curve-fitting term was defined by the RCS function in the rms package. Stratified analyses were used to explore whether these associations remained consistent across subgroups and to explore potential effect modifiers through interaction analyses. In sensitivity analysis, the IDF definition was used to diagnose MetS to verify the stability of the results.

### 2.7 Ethics statement

The NCHS Ethics Review Board reviewed and approved all NHANES protocols, and all participants provided written informed consent. NHANES is a publicly accessible database, and our study was a secondary analysis based on pre-existing data, so local ethical approval was waived.

## 3 Results

### 3.1 Baseline characteristics

A total of 8,095 MetS participants were included. The mean age of the participants was 52.427 years. CKD was present in 2006 MetS participants, with a prevalence of 24.8%. Baseline analyses based on OBS quartiles (Q1 < 14; 14 ≤ Q2 < 20; 20 ≤ Q3 < 25; Q4 ≥ 25) indicated that as OBS increased, participants had higher PIR and daily energy intake and were more likely to be non-Hispanic White, non-single, and have an education level above high school. In addition, the prevalence of CVD and CKD progressively decreased with increasing OBS (both *p* < 0.001) (Table 1). In Supplementary Table S2, baseline analyses according to CKD status showed that OBS, dietary OBS, and lifestyle OBS were all significantly lower in CKD patients (all *p* < 0.05).

**Table 1:** Baseline analysis according to OBS quartiles, NHANES 1999–2018.

Variables	Total (*n* = 8,095)	Q1 (*n* = 2,145)	Q2 (*n* = 2,194)	Q3 (*n* = 1880)	Q4 (*n* = 1876)	*P-*value
Age, year	52.427 ± 0.259	51.867 ± 0.444	52.831 ± 0.403	52.290 ± 0.477	52.640 ± 0.419	0.339
PIR	3.077 ± 0.036	2.609 ± 0.050	3.059 ± 0.054	3.216 ± 0.050	3.391 ± 0.057	<0.0001
Energy intake, kcal/day	2119.527 ± 12.056	1506.959 ± 17.712	1929.829 ± 18.791	2291.244 ± 26.424	2720.442 ± 26.943	<0.0001
OBS.dietary	16.463 ± 0.116	7.611 ± 0.074	14.070 ± 0.052	19.252 ± 0.063	24.477 ± 0.066	<0.0001
OBS.lifestyle	3.673 ± 0.024	3.118 ± 0.033	3.636 ± 0.037	3.741 ± 0.040	4.158 ± 0.036	<0.0001
Sex						0.784
Male	4,041(51.533)	1,146(52.696)	1,074(51.043)	927(51.824)	894(50.708)	
Female	4,054(48.467)	999(47.304)	1,120(48.957)	953(48.176)	982(49.292)	
Race						<0.0001
Mexican American	1,407(7.247)	342(6.429)	349(6.582)	339(7.478)	377(8.490)	
Non-Hispanic Black	1,348(7.818)	490(11.946)	394(8.316)	248(6.219)	216(5.033)	
Non-Hispanic White	4,170(75.316)	1,053(72.431)	1,129(75.460)	1,000(75.764)	988(77.382)	
Other Hispanic	597(4.169)	148(4.467)	179(4.609)	129(3.539)	141(4.032)	
Other Race	573(5.449)	112(4.727)	143(5.032)	164(7.000)	154(5.062)	
Marital Status						0.006
Non-single	5,253(69.019)	1,326(65.510)	1,417(69.946)	1,222(68.089)	1,288(72.147)	
Single	2,842(30.981)	819(34.490)	777(30.054)	658(31.911)	588(27.853)	
Education						<0.0001
<High school	807(4.505)	301(6.932)	230(4.638)	147(3.549)	129(3.054)	
High school	3,242(38.651)	988(47.882)	898(39.909)	708(36.342)	648(31.047)	
>High school	4,046(56.844)	856(45.186)	1,066(55.453)	1,025(60.109)	1,099(65.899)	
Diabetes						0.682
No	5,266(71.743)	1,338(70.860)	1,420(71.000)	1,243(72.346)	1,265(72.771)	
Yes	2,829(28.257)	807(29.140)	774(29.000)	637(27.654)	611(27.229)	
Hypertension						0.405
No	2,766(37.376)	682(38.240)	745(36.987)	682(38.734)	657(35.687)	
Yes	5,329(62.624)	1,463(61.760)	1,449(63.013)	1,198(61.266)	1,219(64.313)	
CVD						<0.001
No	6,749(86.125)	1,681(82.658)	1833(85.734)	1,604(88.329)	1,631(87.601)	
Yes	1,346(13.875)	464(17.342)	361(14.266)	276(11.671)	245(12.399)	
CKD						<0.001
No	6,089(79.957)	1,525(76.657)	1,633(79.022)	1,458(81.751)	1,473(82.261)	
Yes	2006(20.043)	620(23.343)	561(20.978)	422(18.249)	403(17.739)	

Continuous variables were expressed as mean ± standard error and categorical variables were expressed as number (percentage). Continuous variables were compared using weighted ANOVA, and categorical variables were analyzed using chi-square tests.

### 3.2 Association of OBS with CKD prevalence in the MetS population

Table 2 presented the results of multivariate logistic regression analysis. In Model 1 and Model 2, OBS, dietary OBS, and lifestyle OBS were all inversely associated with CKD. In fully adjusted Model 3, each incremental score of OBS, dietary OBS, and lifestyle OBS was associated with a 2% (OR = 0.980, 95% CI = 0.966–0.995, *p* = 0.0084), 1.7% (OR = 0.983, 95% CI = 0.967–0.999, *p* = 0.0351), and 7.3% (OR = 0.927, 95% CI = 0.880–0.976, *p* = 0.0049) reduction in the prevalence of CKD. Higher OBS, dietary OBS, and lifestyle OBS were all associated with significantly lower CKD prevalence (*p* for trend = 0.0062, 0.0291, and 0.0028, respectively).

**Table 2:** Association of OBS with CKD in the MetS population, NHANES 1999–2018.

	Model 1 OR (95%CI) *p*-value	Model 2 OR (95%CI) *p*-value	Model 3 OR (95%CI) *p*-value
OBS	0.976 (0.966, 0.986) <0.0001	0.977 (0.967, 0.988) <0.0001	**0.980 (0.966, 0.995) 0.0084**
OBS quartile			
Q1	Ref.	Ref.	Ref.
Q2	0.830 (0.679, 1.015) 0.0720	0.841 (0.689, 1.027) 0.0922	0.875 (0.707, 1.083) 0.2222
Q3	0.701 (0.575, 0.855) 0.0006	0.718 (0.589, 0.875) 0.0013	**0.747 (0.598, 0.934) 0.0115**
Q4	0.636 (0.520, 0.778) <0.0001	0.655 (0.535, 0.801) 0.0001	**0.693 (0.530, 0.905) 0.0081**
*P* for trend	<0.0001	<0.0001	**0.0062**
OBS.DIETARY	0.978 (0.967, 0.989) 0.0001	0.980 (0.969, 0.991) 0.0004	**0.983 (0.967, 0.999) 0.0351**
OBS.DIETARY quartile			
Q1	Ref.	Ref.	Ref.
Q2	0.801 (0.654, 0.981) 0.0338	0.818 (0.668, 1.002) 0.0548	0.863 (0.694, 1.072) 0.1851
Q3	0.669 (0.556, 0.804) <0.0001	0.687 (0.571, 0.826) 0.0001	**0.715 (0.579, 0.881) 0.0021**
Q4	0.683 (0.564, 0.826) 0.0001	0.707 (0.583, 0.858) 0.0006	**0.765 (0.588, 0.997) 0.0492**
*P* for trend	0.0001	0.0004	**0.0291**
OBS.LIFESTYLE	0.904 (0.861, 0.950) 0.0001	0.902 (0.858, 0.948) 0.0001	**0.927 (0.880, 0.976) 0.0049**
OBS.LIFESTYLE quartile			
Q1	Ref.	Ref.	Ref.
Q2	0.852 (0.697, 1.042) 0.1201	0.851 (0.696, 1.039) 0.1147	0.877 (0.715, 1.074) 0.2067
Q3	0.773 (0.633, 0.943) 0.0122	0.766 (0.626, 0.936) 0.0101	0.844 (0.687, 1.038) 0.1107
Q4	0.665 (0.548, 0.806) 0.0001	0.659 (0.543, 0.801) <0.0001	**0.727 (0.596, 0.887) 0.0021**
*P* for trend	<0.0001	<0.0001	**0.0028**

Model 1 did not adjust for any covariates, model 2 partially adjusted for age, sex, and race, and model 3 additionally adjusted for education, PIR, marital status, daily energy intake, diabetes mellitus, hypertension, and CVD on top of model 2. Bold values represent the statistically significant results.

### 3.3 RCS analysis

**Figure 2:**
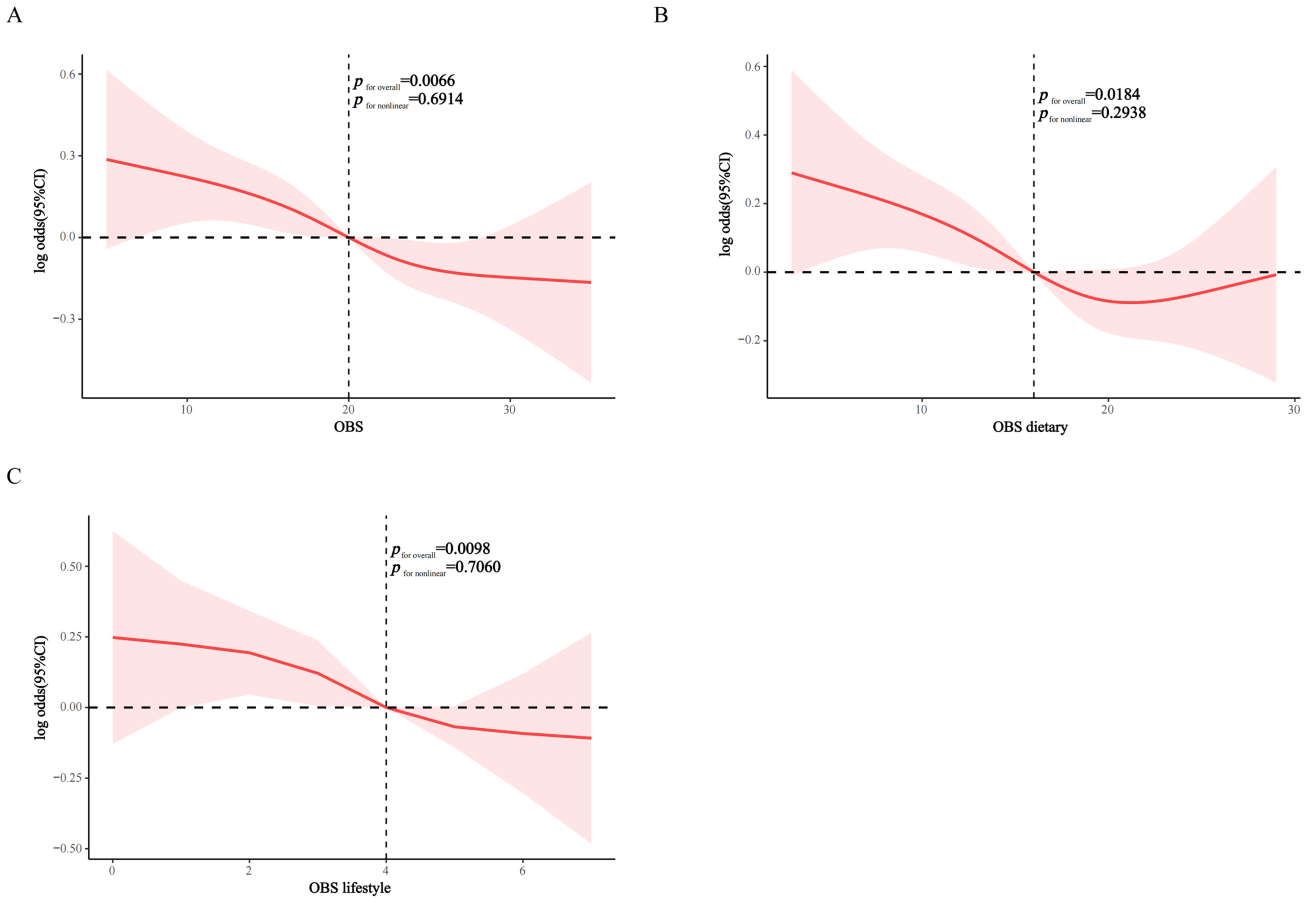
RCS modeling of the association of OBS, dietary OBS, and lifestyle OBS with CKD in the MetS population. **(A)** OBS; **(B)** dietary OBS; **(C)** lifestyle OBS.

RCS analysis demonstrated significant inverse linear associations between OBS, dietary OBS, and lifestyle OBS with CKD prevalence in MetS (*p* for non-linearity = 0.6914, 0.2938, and 0.7060, respectively), suggesting that higher OBS is associated with lower CKD prevalence in MetS in a dose–response manner (Figures 2A–C).

### 3.4 Stratified analysis

**Figure 3:**
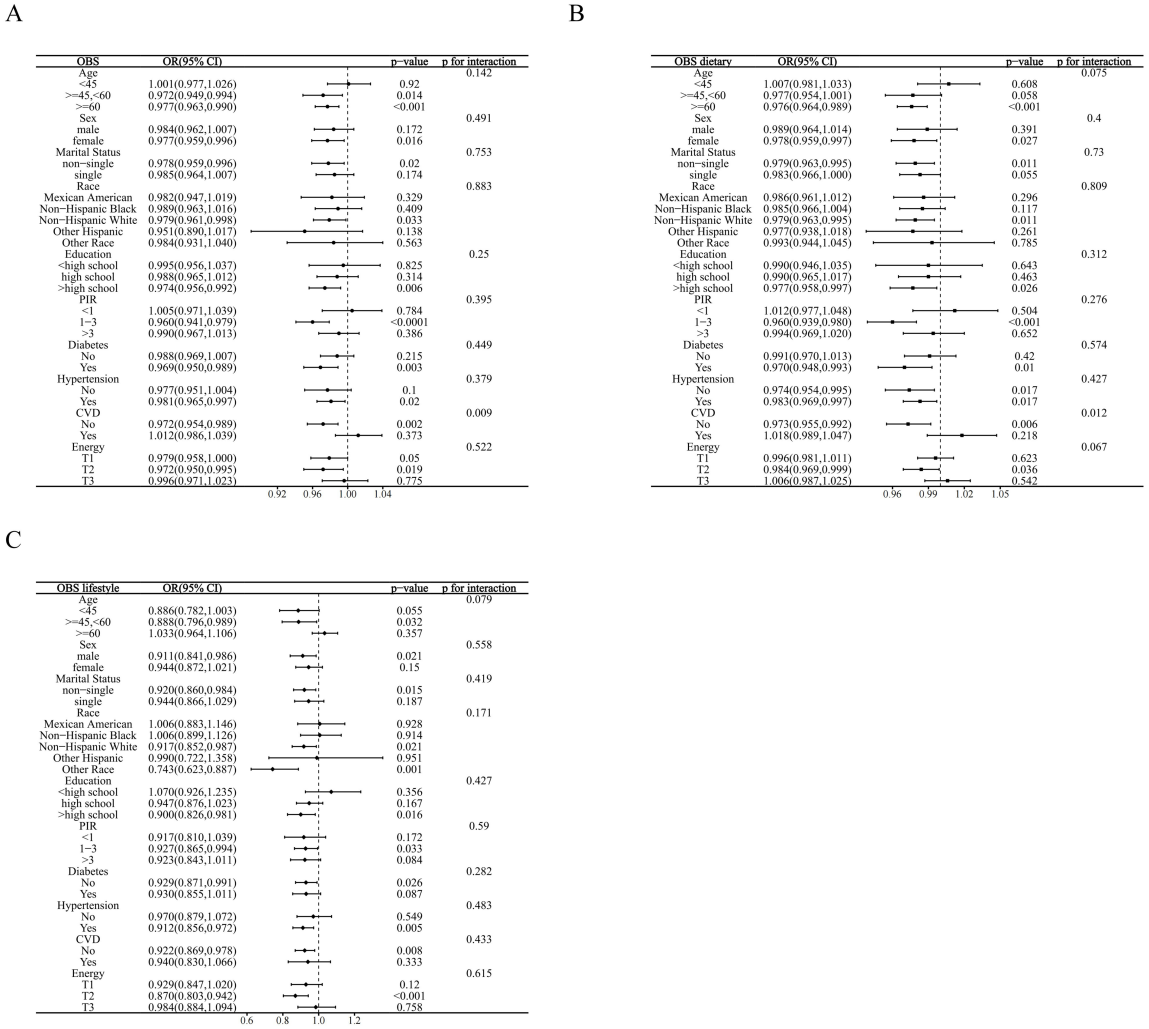
Stratified analysis based on inclusion of covariates. **(A)** OBS; **(B)** dietary OBS; **(C)** lifestyle OBS.

We stratified the associations of OBS, dietary OBS, and lifestyle OBS with CKD according to the included covariates. Most covariates did not significantly affect these associations, however, interaction analyses indicated that CVD was the effect modifier in the associations of OBS and dietary OBS with CKD (*p* for interaction 0.009 and 0.012, respectively). These effects were only present in MetS patients without CVD (OBS: OR = 0.972, *p* = 0.002; dietary OBS: OR = 0.973, *p* = 0.006) and disappeared in CVD co-morbid individuals. Finally, the association between lifestyle OBS and the prevalence of CKD in MetS remained stable across subgroups (all *p* for interaction >0.05) (Figures 3A–C).

### 3.5 Sensitivity analysis

Using the IDF criteria to define MetS did not significantly change the results (Supplementary Table S3), with OBS, dietary OBS, and lifestyle OBS remaining inversely associated with the prevalence of CKD in the MetS population after adjusting for all confounders (OBS: OR = 0.980, *p* = 0.0037; Dietary OBS: OR = 0.982, *p* = 0.0007; Lifestyle OBS: OR = 0.946, *p* = 0.0046), suggesting consistency and robustness of these findings.

## 4 Discussion

Leveraging a nationally representative population-based cross-sectional study, our findings showed for the first time that an emerging composite dietary and lifestyle oxidative balance assessment metric, OBS, was significantly and negatively associated with the prevalence of CKD in the MetS population. Each one-point increase in overall OBS, dietary OBS, and lifestyle OBS in MetS individuals was associated with a 2, 1.7, and 7.3% lower prevalence of CKD, respectively, and all exhibited dose–response patterns. CVD co-morbidity in MetS significantly affected the association between OBS and dietary OBS and CKD, suggesting the existence of an interaction between MetS, CVD, and CKD. These findings emphasize that the antioxidant potential of dietary and lifestyle sources may confer an important protective role against the development of CKD in MetS. Adherence to an OBS-assessed antioxidant diet and lifestyle, which are modifiable risk factors, all contribute to the prevention of CKD, especially in the CVD-free cohort. Our findings may provide new insights for CKD prevention in MetS in policy making and public health.

The OBS is an emerging composite metric for assessing an individual’s overall redox homeostasis that integrates antioxidant and pro-oxidant exposures from diet and lifestyle. By evaluating the impact of overall diet and lifestyle on oxidative stress, higher OBS suggests higher antioxidant exposure. In the organism, a multifactorial imbalance of pro-oxidants and antioxidants may lead to oxidative stress, which can initiate cellular damage and death. A large body of evidence suggests that diet and lifestyle may influence the intrinsic oxidative stress process by modulating oxidative homeostasis (28–30). However, assessing the effects of individual antioxidants and pro-oxidants on health and disease has produced inconsistent results in previous clinical studies, and a possible explanation for this is that antioxidant and pro-oxidant interactions may have been overlooked (19). Therefore, integrating dietary and lifestyle influences on oxidative stress may more accurately reflect overall antioxidant exposure. The first OBS consists of two dietary antioxidants (vitamin C and beta carotene) and one pro-oxidant (iron) (31). Extensive subsequent epidemiologic studies have continually updated and expanded the components of OBS, and there are now more than 20 types of OBS with different assignment modes and components (19). In this study, we used the OBS assessment criteria that were extensively validated in previous NHANES-related studies, which consisted of 16 dietary components and 4 lifestyle components that comprehensively considered the complex effects of antioxidants and pro-oxidants (22, 23, 32, 33). Thus, our findings suggest that higher antioxidant properties of both diet and lifestyle are associated with lower prevalence of CKD in MetS, indicating independent and joint associations.

A large body of experimental evidence suggests that oxidative stress is an important contributor in both the pathogenesis and disease progression of MetS and CKD (34, 35). Accumulating evidence also suggests a key pathophysiological role for oxidative stress in the development and progression of MetS-related CKD. Adipose tissue dysfunction and aberrant secretion and release of adipokines and inflammatory cytokines in MetS may induce systemic chronic low-grade inflammation, oxidative stress, and endothelial dysfunction, resulting in renal injury and dysfunction (12, 36). Increased levels of reactive oxygen species and oxidative stress in MetS are responsible for renal microvascular remodeling and structural/functional alterations by inducing endothelial dysfunction and hypercoagulability, which may be important pathophysiological events in the development and progression of CKD (14, 15).

Previous clinical findings or experimental studies suggest that dietary antioxidants and physical activity may prevent the development of MetS and CKD (18, 37), whereas pro-oxidants such as high-fat diets (38), smoking (39, 40), and excessive alcohol consumption (6, 41) may promote the development of these conditions. However, the association of these antioxidants and pro-oxidants with the prevalence of CKD in MetS remains understudied. Furthermore, to the best of our knowledge, there are no studies addressing the association of OBS with the prevalence of CKD in MetS. However, several clinical studies have shown that OBS is associated with the development and progression of several diseases, including MetS and CKD, and inconsistent conclusions exist. A previous cross-sectional study that included 847 Iranian participants showed that OBS was unrelated to MetS after adjusting for confounders (*p* = 0.07) (42). A subsequent cross-sectional analysis and cohort study from Korea demonstrated that OBS was negatively associated with both the prevalence and incidence of MetS (43). Cross-sectional analyses using NHANES similarly showed that OBS was negatively associated with MetS prevalence, MetS severity, and all-cause mortality from MetS (23, 44). Several previous cohort studies have explored the association of OBS with CKD in the general population. Ilori et al. (20) suggested that OBS was inversely associated with baseline CKD prevalence in a large cohort study that included 19,461 US participants (*p* for trend <0.01). A recent cohort study from Korea similarly demonstrated that OBS was negatively associated with incident CKD in both men and women (21). However, another cohort study that included 3,233 participants with CKD suggested OBS was not associated with end-stage renal disease in the fully adjusted model (45). As mentioned previously, these studies had relatively few OBS components (13 and 15) and did not independently explore the effects of dietary OBS and lifestyle OBS on CKD. Our study provides new insights into the prevention of CKD by OBS in individuals with MetS and suggests that both adherence and antioxidant diet and lifestyle have inverse dose–response associations with CKD prevalence. A diet rich in anti-inflammatory nutrients, fiber, and phytochemicals has been demonstrated to mitigate proteinuria, decelerate the progression of CKD, and postpone the necessity for dialysis. This can be achieved by reducing cardiometabolic risk factors such as hypertension, CVD, diabetes, and obesity (46, 47). Therefore, patients with CKD are advised to increase the proportion of plant-based foods and reduce the intake of animal-based foods (e.g., those in the Mediterranean diet pattern) as recommended by the 2024 KDIGO practice guideline (48). In addition to dietary modifications, patients are encouraged to perform moderate-intensity physical activity for at least 150 min/week or an amount that is appropriate for their cardiovascular and physical fitness, maintain an optimal BMI, and avoid tobacco products.

Another important finding of our study was that CVD significantly influenced the impact of OBS and dietary OBS on CKD in the MetS population, but not lifestyle OBS. These associations were only present in CVD-free individuals with MetS, suggesting that the presence of CVD may diminish the protective benefits of OBS. Interactions between MetS, CKD, and CVD have been well documented. As three major global public health concerns, the recently proposed CKM syndrome links CKD, CVD, and metabolic disorders, suggesting that these diseases require multidisciplinary integrated management approaches (9–11). The pathophysiologic mechanisms of CKM syndrome are thought to be complex and multifaceted, and oxidative stress may play an important contributory role (9, 49). Adherence to a healthy diet and physically active lifestyle are important strategies for CKM syndrome prevention, especially in the early stages (9). Our study consistently indicates the existence of MetS, CKD, and CVD interactions. Importantly, our study reveals for the first time the protective role of adherence to an antioxidant diet against CKD in MetS, especially in CVD-free populations.

Our research strengths are reflected in the following aspects. First, this is a large, nationally representative population-based cross-sectional analysis, and its large sample and multiethnicity make these findings well generalizable and replicable. The OBS calculation was based on assessment criteria that have been extensively validated in previous NHANES-related studies, and fully considered dietary and lifestyle antioxidants and pro-oxidants to reflect an individual’s oxidative homeostasis more accurately. Our study adequately considered potential confounding factors and reduced the confounding bias of the study. However, there are limitations to our study. First, it is a cross-sectional analysis, and therefore cannot draw causal associations and may still be subject to residual confounders. Future prospective cohort studies are needed to confirm these findings. Second, dietary intake was based on participants’ self-reports, which may be subject to recall bias. However, previous NHANES-related studies have similarly demonstrated the reliability and consistency of these questionnaires (50). In addition, we were unable to explore the effect of OBS on the progression of CKD across different staging and disease stages in MetS. Future studies are needed to explore the longitudinal association between OBS and CKD progression in MetS.

## 5 Conclusion

In a national cross-sectional analysis, overall OBS, dietary OBS, and lifestyle OBS were all inversely associated with the prevalence of CKD among patients with MetS, and all had dose–response associations. CVD significantly influenced the association of OBS and dietary OBS with the prevalence of CKD in the MetS population, and these effects were only present in CVD-free individuals. These findings suggest that compliance with an antioxidant diet and lifestyle as assessed by OBS both contribute to the prevention of CKD in individuals with MetS and support relevant policy development and public health focus.

## Data Availability

This study analyzed publicly available datasets and can be found at https://www.cdc.gov/nchs/nhanes/.
