# Supplementary material for: Higher oxidative balance score is linearly associated with reduced prevalence of chronic kidney disease in individuals with metabolic syndrome: evidence from NHANES 1999–2018
Source: Front Nutr. 2024 Sep 30;11:1442274. doi: 10.3389/fnut.2024.1442274 (PMC11472227; doi:10.3389/fnut.2024.1442274)
Supplement: Supplementary file 1 [file Table_1.DOCX]

**Table S1**. Assignment criteria and component details for OBS.

|  |  | Male |  |  | Female |  |  |
| --- | --- | --- | --- | --- | --- | --- | --- |
|  | Property | 0 | 1 | 2 | 0 | 1 | 2 |
| Dietary OBS components |  |  |  |  |  |  |  |
| Dietary fiber (g/d) | A | <12.55 | 12.5-19.67 | >19.67 | <10.05 | 10.05-16.33 | >16.33 |
| Carotene (RE/d) | A | <98.62 | 98.62-305.85 | >305.85 | <98.06 | 98.06-383.92 | >383.92 |
| Riboflavin (mg/d) | A | <1.79 | 1.79-2.69 | >2.69 | <1.34 | 1.34-2.02 | >2.02 |
| Niacin (mg/d) | A | <20.64 | 20.64-29.75 | >29.75 | <14.52 | 14.52-21.85 | >21.85 |
| Vitamin B6 (mg/d) | A | <1.59 | 1.59-2.40 | >2.40 | <1.13 | 1.13-1.77 | >1.77 |
| Total folate (mcg/d) | A | <315.52 | 315.52-491.5 | >491.5 | <250.50 | 250.50-388.5 | >388.5 |
| Vitamin B12 (mcg/d) | A | <3.35 | 3.35-6.20 | >6.20 | <2.22 | 2.22-4.21 | >4.21 |
| Vitamin C (mg/d) | A | <42.40 | 42.40-113.15 | >113.15 | <38.01 | 38.01-98.40 | >98.40 |
| Vitamin E (ATE) (mg/d) | A | <5.82 | 5.82-9.41 | >9.41 | <4.53 | 4.53-7.52 | >7.52 |
| Calcium (mg/d) | A | <645.50 | 645.50-1072.50 | >1072.50 | <499.00 | 499.00-848.50 | >848.50 |
| Magnesium (mg/d) | A | <256.50 | 256.50-361.02 | >361.02 | <186.53 | 186.53-283.00 | >283.00 |
| Zinc (mg/d) | A | <9.74 | 9.74-15.10 | >15.10 | <6.73 | 6.73-10.74 | >10.74 |
| Copper (mg/d) | A | <1.12 | 1.12-1.57 | >1.57 | <0.85 | 0.85-1.28 | >1.28 |
| Selenium (mcg/d) | A | <94.90 | 94.90-141.75 | >141.75 | <67.75 | 67.75-99.5 | >99.5 |
| Total fat (g/d) | P | >107.42 | 69.83-107.42 | <69.83 | >75.73 | 50.99-75.73 | <50.94 |
| Iron (mg/d) | P | >19.17 | 12.87-19.17 | <12.87 | >14.32 | 9.65-14.32 | <9.65 |
| Lifestyle OBS components |  |  |  |  |  |  |  |
| Physical activity (MET-minute/week) | A | <415.68 | 415.68-1134.00 | >1134.00 | <264.13 | 264.13-843.56 | >843.56 |
| Alcohol (drinks/d) | P | >30g/day | 0-30g/day | None | >15g/day | 0-15g/day | None |
| Body mass index (kg/m^2^) | P | >29.17 | 25.54-29.17 | <25.54 | >28.63 | 23.74-28.63 | <23.74 |
| Cotinine (ng/mL) | P | >1.13 | 0.04-1.13 | <0.04 | >0.17 | 0.04-0.17 | <0.04 |

A: antioxidant; P: pro-oxidant.
