# Supplementary material for: Higher oxidative balance score is linearly associated with reduced prevalence of chronic kidney disease in individuals with metabolic syndrome: evidence from NHANES 1999–2018
Source: Front Nutr. 2024 Sep 30;11:1442274. doi: 10.3389/fnut.2024.1442274 (PMC11472227; doi:10.3389/fnut.2024.1442274)
Supplement: Supplementary file 2 [file Table_2.DOCX]

**Table S2**. Baseline analysis according to CKD status.

|  | **Total (n=8095)** | **No-CKD (n=6089)** | **CKD (n=2006)** | **P value** |
| --- | --- | --- | --- | --- |
| **Age, year** | 52.427±0.259 | 50.246±0.261 | 61.125±0.487 | < 0.0001 |
| **PIR** | 3.077±0.036 | 3.145±0.038 | 2.802±0.053 | < 0.0001 |
| **Energy intake, kcal/day** | 2119.527±12.056 | 2165.985±13.362 | 1934.193±25.586 | < 0.0001 |
| **OBS.dietary** | 16.463±0.116 | 16.674±0.128 | 15.622±0.215 | < 0.0001 |
| **OBS.lifestyle** | 3.673±0.024 | 3.704±0.026 | 3.528±0.042 | < 0.0001 |
| **OBS** | 20.137±0.126 | 20.334±0.141 | 19.350±0.221 | < 0.001 |
| **Sex** |  |  |  | 0.028 |
| male | 4041(51.533) | 3022(52.246) | 1019(48.686) |  |
| female | 4054(48.467) | 3067(47.754) | 987(51.314) |  |
| **Race** |  |  |  | 0.003 |
| Mexican American | 1407(7.247) | 1084(7.368) | 323(6.764) |  |
| Non-Hispanic Black | 1348(7.818) | 970(7.267) | 378(10.018) |  |
| Non-Hispanic White | 4170(75.316) | 3114(75.782) | 1056(73.459) |  |
| Other Hispanic | 597(4.169) | 480(4.207) | 117(4.018) |  |
| Other Race | 573(5.449) | 441(5.375) | 132(5.741) |  |
| **Marital Status** |  |  |  | < 0.0001 |
| non-single | 5253(69.019) | 4082(70.527) | 1171(63.005) |  |
| single | 2842(30.981) | 2007(29.473) | 835(36.995) |  |
| **Education** |  |  |  | < 0.0001 |
| <high school | 807(4.505) | 547(3.832) | 260(7.192) |  |
| high school | 3242(38.651) | 2409(38.115) | 833(40.789) |  |
| >high school | 4046(56.844) | 3133(58.053) | 913(52.019) |  |
| **Diabetes** |  |  |  | < 0.0001 |
| No | 5266(71.743) | 4320(76.522) | 946(52.680) |  |
| Yes | 2829(28.257) | 1769(23.478) | 1060(47.320) |  |
| **Hypertension** |  |  |  | < 0.0001 |
| No | 2766(37.376) | 2384(41.636) | 382(20.379) |  |
| Yes | 5329(62.624) | 3705(58.364) | 1624(79.621) |  |
| **CVD** |  |  |  | < 0.0001 |
| No | 6749(86.125) | 5321(88.899) | 1428(75.056) |  |
| Yes | 1346(13.875) | 768(11.101) | 578(24.944) |  |

Continuous variables were expressed as mean ± standard error and categorical variables were expressed as number (percentage). Continuous variables were compared using weighted t tests, and categorical variables were analyzed using chi-square tests.
