# Supplementary material for: Higher oxidative balance score is linearly associated with reduced prevalence of chronic kidney disease in individuals with metabolic syndrome: evidence from NHANES 1999–2018
Source: Front Nutr. 2024 Sep 30;11:1442274. doi: 10.3389/fnut.2024.1442274 (PMC11472227; doi:10.3389/fnut.2024.1442274)
Supplement: Supplementary file 3 [file Table_3.DOCX]

**Table S3**. Sensitivity analysis using IDF criteria for diagnosis of MetS.

|  | **Model 1**  **OR (95%CI) P-value** | **Model 2**  **OR (95%CI) P-value** | **Model 3**  **OR (95%CI) P-value** |
| --- | --- | --- | --- |
| **OBS** | 0.977 (0.970, 0.984) <0.0001 | 0.978 (0.971, 0.985) <0.0001 | 0.980 (0.971, 0.990) 0.0037 |
| **OBS quartile** |  |  |  |
| Q1 | Ref. | Ref. | Ref. |
| Q2 | 0.813 (0.708, 0.935) 0.0036 | 0.821 (0.714, 0.944) 0.0054 | 0.858 (0.740, 0.996) 0.0434 |
| Q3 | 0.752 (0.651, 0.868) 0.0001 | 0.767 (0.664, 0.887) 0.0003 | 0.795 (0.674, 0.938) 0.0065 |
| Q4 | 0.643 (0.559, 0.740) <0.0001 | 0.656 (0.569, 0.756) <0.0001 | 0.696 (0.578, 0.838) 0.0001 |
| **P for trend** | <0.0001 | <0.0001 | 0.0007 |
| **OBS.DIETARY** | 0.979 (0.971, 0.986) <0.0001 | 0.980 (0.972, 0.987) <0.0001 | 0.982 (0.971, 0.992) 0.0007 |
| **OBS.DIETARY quartile** |  |  |  |
| Q1 | Ref. | Ref. | Ref. |
| Q2 | 0.780 (0.677, 0.897) 0.0005 | 0.787 (0.684, 0.906) 0.0008 | 0.826 (0.711, 1.060) 0.1280 |
| Q3 | 0.740 (0.644, 0.851) 0.0002 | 0.752 (0.654, 0.866) 0.0001 | 0.773 (0.659, 0.907) 0.0015 |
| Q4 | 0.677 (0.591, 0.775) <0.0001 | 0.689 (0.601, 0.790) <0.0001 | 0.729 (0.607, 0.875) 0.0007 |
| **P for trend** | <0.0001 | <0.0001 | 0.0034 |
| **OBS.LIFESTYLE** | 0.912 (0.879, 0.946) <0.0001 | 0.917 (0.884, 0.952) <0.0001 | 0.946 (0.910, 0.983) 0.0046 |
| **OBS.LIFESTYLE quartile** |  |  |  |
| Q1 | Ref. | Ref. | Ref. |
| Q2 | 0.898 (0.765, 1.055) 0.1909 | 0.907 (0.772, 1.066) 0.2377 | 0.932 (0.790, 1.099) 0.4024 |
| Q3 | 0.887 (0.757, 1.040) 0.1399 | 0.897 (0.764, 1.052) 0.1809 | 0.969 (0.822, 1.142) 0.7052 |
| Q4 | 0.703 (0.603, 0.819) <0.0001 | 0.718 (0.615, 0.839) 0.0003 | 0.797 (0.679, 0.936) 0.0055 |
| **P for trend** | <0.0001 | <0.0001 | 0.0046 |

Model 1 did not adjust for any covariates, model 2 partially adjusted for age, sex, and race, and model 3 additionally adjusted for education, PIR, marital status, daily energy intake, diabetes mellitus, hypertension, and CVD on top of model 2.
